# Global Prioritization of Disease Candidate Metabolites Based on a Multi-omics Composite Network

**DOI:** 10.1038/srep17201

**Published:** 2015-11-24

**Authors:** Qianlan Yao, Yanjun Xu, Haixiu Yang, Desi Shang, Chunlong Zhang, Yunpeng Zhang, Zeguo Sun, Xinrui Shi, Li Feng, Junwei Han, Fei Su, Chunquan Li, Xia Li

**Affiliations:** 1 College of Bioinformatics Science and Technology, Harbin Medical University, Harbin, 150081, China; 2 School of Medical Informatics, Daqing Campus, Harbin Medical University, 39 Xinyang Road, Harbin 163319, China

## Abstract

The identification of disease-related metabolites is important for a better understanding of metabolite pathological processes in order to improve human medicine. Metabolites, which are the terminal products of cellular regulatory process, can be affected by multi-omic processes. In this work, we propose a powerful method, MetPriCNet, to predict and prioritize disease candidate metabolites based on integrated multi-omics information. MetPriCNet prioritized candidate metabolites based on their global distance similarity with seed nodes in a composite network, which integrated multi-omics information from the genome, phenome, metabolome and interactome. After performing cross-validation on 87 phenotypes with a total of 602 metabolites, MetPriCNet achieved a high AUC value of up to 0.918. We also assessed the performance of MetPriCNet on 18 disease classes and found that 4 disease classes achieved an AUC value over 0.95. Notably, MetPriCNet can also predict disease metabolites without known disease metabolite knowledge. Some new high-risk metabolites of breast cancer were predicted, although there is a lack of known disease metabolite information. A predicted disease metabolic landscape was constructed and analyzed based on the results of MetPriCNet for 87 phenotypes to help us understand the genetic and metabolic mechanism of disease from a global view.

Metabolites, which are the terminal products of cellular regulatory process, are usually considered the ultimate response of biological systems to changes of inheritance or environment^1^. Metabolite levels can directly reflect the physiological state of the human body. Identifying disease-related metabolites is highly significant not only for improving clinical diagnosis but also for a better understanding of metabolic pathological processes^1^,^2^,^3^. With the development of metabolomics technology, hundreds to thousands of metabolites can be detected by gas chromatography-mass spectrometry (GC-MS), liquid chromatography-mass spectrometry (LC-MS), nuclear magnetic resonance (NMR) and other technologies^4^. Identifying and prioritizing high-risk disease metabolites is becoming a challenging task.

Metabolites rarely function in isolation, but as the link between genotypes and phenotypes, they are always influenced by the joint power of genome and phenome^5^. The impact of one disease is not restricted to one or two metabolites but spreads among functionally related metabolites and genes that organize into an intricate network. Thus, functionally related metabolites and genes tend to relate to phenotypically similar diseases. The development of various ‘omics’ data, such as metabolic, genomic and phenomic, will provide valuable information for disease risk candidate metabolites prioritization. “Multi-omics” integration strategies can provide deeper mechanistic insight into biological systems. From a biological point of view, a biological system can be presented as a multi-omics network. Integrating gene, metabolite and phenotype information is a natural way to construct a composite network for identifying disease metabolites, and this integration strategy can provide comprehensive and accurate information^6^,^7^. Although metabolomics has developed rapidly, there is still bias in metabolite identification, and the current metabolic data are not sufficient^8^. At the same time, the discovery of disease-related genes is more mature. Compared with 190 OMIM phenotypes associated with 877 known disease metabolites listed in HMDB version 2.5, there are over five thousand phenotypes and over three thousand genes listed in the current OMIM database. Merging gene and phenotype information to construct a composite network could provide comprehensive information to identify disease metabolites. If some information is not available, the integration strategy could use other information to compensate for the missing information. We previously developed a method (PROFANCY) to prioritize disease metabolites based on the context of the metabolite network (or metabolite pathways)^9^. However, some other omics interactions (for example, phenotype-gene interactions, gene-gene interactions and gene-metabolite interactions) and known disease information have been largely ignored. When disease metabolite information is lacking, PROFANCY loses its efficiency. Some integration methods have been proposed to solve this limit in disease gene prioritization. Zhao and Chen *et al.* integrated mRNA expression and pathway interaction information to identify cancer-related miRNA and dysregulated genes (pathways)^10^,^11^. Wu *et al.* developed CIPHER, which integrates the phenome and protein-protein interactome to capture the relationships between phenotypes and genotypes to predict disease genes, and achieved high prediction power^12^,^13^. However, their “dual-omics” network was not sufficient for disease metabolite candidate prioritization because metabolites were considered as terminal products of complex cellular regulatory processes. Therefore, it’s also likely to be available to integrated multi-omics information to prioritize disease risk metabolites.

In this work, we proposed a computational method, MetPriCNet (disease candidate Metabolites Prioritization based on the Composite Network through integrating multi-omics data), to predict and prioritize disease risk metabolites. We constructed a composite network that integrated multi-omics data, including genome, phenome, metabolome and interactome. Based on the global functional relations of the composite network, the candidate metabolites associated with a certain phenotype were prioritized. MetPriCNet had high prediction power not only with respect to overall performance but also for different disease classes. Notably, we demonstrate its effectiveness for diseases without known disease metabolites by a metabolome-wide scan of disease metabolites. For the prioritization of prostate cancer metabolites, sarcosine, a metabolite recently discovered to be important in invasion and aggressive progression, was ranked number 1 in MetPriCNet. We also delineate a metabolomic landscape to investigate diseases from a global perspective. MetPriCNet is freely available at http://www.bio-bigdata.net/MetPriCNet/.

## Material and Methods

### Data sets

The known disease metabolites were extracted from the Human Metabolome Database (HMDB)^14^. HMDB collects detailed information of small-molecule metabolites of human and their disease phenotype information described in the entries in OMIM. The known disease gene information was obtained from the Morbid Map file from OMIM database^15^, which contains comprehensive, curated descriptions of human genes and phenotypes and the relationships between them.

We constructed a weighted composite network by integrating six data sets, which can be represented by six networks, namely i) gene network, ii) metabolite network, iii) phenotype network, iv) gene-metabolite association network, v) phenotype-gene association network, and vi) phenotype-metabolite association network. We define i) – iii) as the basic network and iv) – vi) as the interaction network. The detailed information is described in the following sections.

1. The gene network is composed of 1,515,370 gene-gene associations and their confidence scores between 16,785 human genes. These interactions are obtained according to the proteins they encode interact with each other in the STRING database^16^, which provides comprehensive coverage of both the experimental and predicted interaction information and confidence scores for each interaction.
2. To construct the metabolite network, we first collected 4,994 human metabolites from metabolite pathways of KEGG^17^ and HMDB^14^, and the human pathways of Reactome^18^, MSEA^19^ and SMPDB^20^. Then, we extracted human metabolite-metabolite associations and their confidence scores from STITCH^21^, in which the metabolite must be contained in the 4,994 human metabolites. There are four types of chemical-chemical associations in STITCH, including reactions from pathway databases, literature associations, similar chemical structures and similar molecular activities. We obtained 74,667 human metabolite-metabolite associations and their confidence scores in 3,764 human metabolites (not all metabolites have associations in STITCH).
3. The phenotype network was constructed using the phenotype-phenotype similarity associations from van Driel *et al.*^22^, which stores 5,080 phenotypes and the similarity scores among them. These phenotypes cover the majority of recorded human phenotypes.
4. To extract human gene-metabolite associations, we extracted the chemical and human gene associations and their confidence scores from STITCH. Then, we extracted human metabolite and gene associations according to the 4,994 human metabolites. After filtering the metabolites that are not involved in our metabolite network and the genes that are not included in the gene network, we obtained 192,763 gene-metabolite associations, including 12,342 genes and 3,278 metabolites.
5. We obtained phenotype-gene associations from the curated Morbid Map file from OMIM. There were 2,603 associations between 1,886 phenotypes and 1,715 genes after filtering the phenotypes not in our phenotype network and the genes not in our gene network. We define the weighted score as 1 for each phenotype-gene association.
6. Phenotype-metabolite associations were obtained from HMDB. Similarly, 664 phenotype-metabolite associations between 149 phenotypes and 388 metabolites remained after filtration. We define the weighted score as 1 for each phenotype-metabolite association.

### MetPriCNet

**Figure 1:**
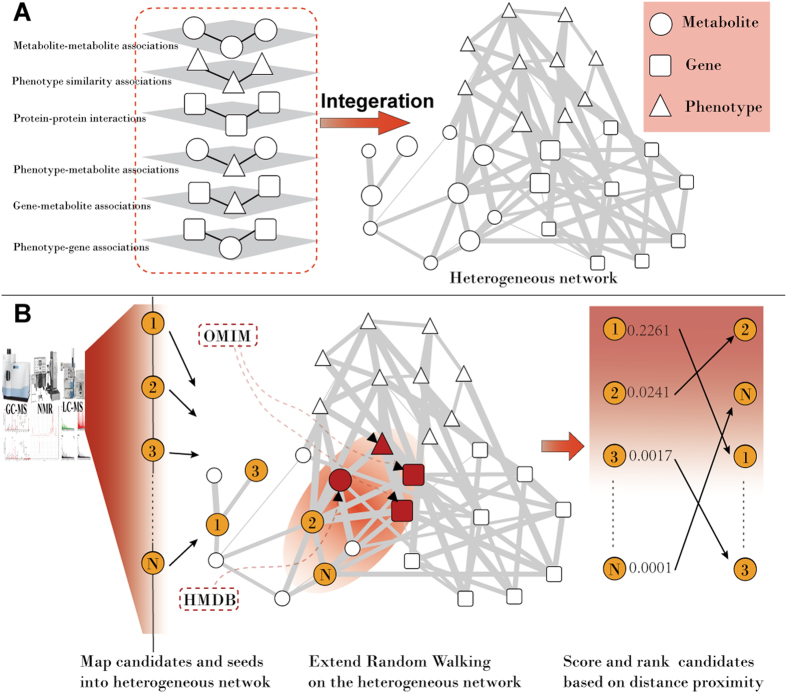
The flow chart of MetPriCNet. (**A**) Construction of the multi-omics composite network. The multi-omics composite network is composed of six sub-networks. White circles indicate metabolites, white squares indicate genes, and white triangles indicate phenotypes. The thickness of an edge indicates the weight score. (**B**) The flow chart of MetPriCNet to optimize the candidate metabolite. First, the interested candidate metabolite and seed nodes are mapped to the multi-omics composite network. Then, a global extended RWR method is used to score the candidate metabolites according to their proximity to the seed nodes. Finally, the candidate metabolites are ranked by the scores. Orange circles represent the candidate metabolites of interest. Red triangles indicate the disease phenotype of interest (phenotype seed) from the OMIM data base, red squares represent known disease genes (gene seeds) from the OMIM database, and red circles indicate known disease metabolites (metabolite seeds) from the HMDB database.

The general idea of MetPriCNet is depicted in Fig. 1. First, we constructed a composite network, which integrated the omics data, including the genome, phenome, metabolome and interactome. Then, we proposed a global computational method to capture the interaction information between the multi-omics composite network to prioritize the candidate metabolites according to their proximity with known disease seed nodes. The details are given below.

### The composite network construction

To construct a multi-omics composite network, the six networks described above in the data sets were integrated to one weighted composite network. Let 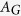, 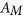, 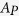, 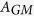, 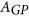, 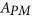 be the adjacency matrix of the above six networks i) – vi), respectively. Then, the adjacency matrix of the composite network can be written as 
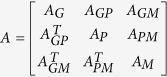
, where the right corner of the adjacency matrix T represents the transpose of it.

### Candidate metabolite prioritization based on the composite network

To capture the global omics information of the composite network for candidate metabolite prioritization, we extended the random walk with restart (RWR) method, introduced by Kohler, S *et al.*^12^, to a multi-omics composite network. The method prioritizes candidate metabolites based on the proximity of each candidate to the seed node(s) in the network and simulates a random walker starting on a seed node(s). At each step, the walker moves from the current node(s) to its immediate neighbors with probability 1 − *α* or returns to the seed node(s) with probability *α*. In this method, let 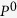 be the initial probability vector and 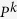 represent a vector in which the 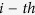 element holds the probability of being at node *i* at step *k*. Then, the probability 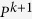 is defined as


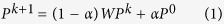


where *W* is the transition matrix of the composite network, which is a column-normalized adjacency matrix of the composite network and can be inferred from the adjacency matrix *A*. The detailed information will be introduced below. After multiple step iterations, the probability reaches a steady state, and the iterations stop until the change between 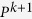 and 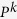 falls below 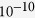 (measured by the L1 norm).

In this study, to construct the transition matrix, let 
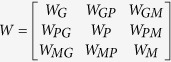
. 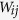 represents the probability from node *i* to node *j*. Let *x*, *y*, *z* be the jumping probability between the gene network and the phenotype network, the gene network and the metabolite network, and the phenotype network and the metabolite network, respectively. Then, the probability from gene *i* (*g*_*i*_) to gene *j* (*g*_*j*_) can be computed as follows:


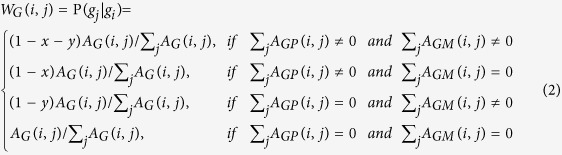


Similarly, the transition probability from gene *i* (*g*_*i*_) to phenotype *j* (*p*_*j*_) can be defined as


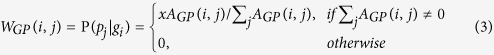


The transition probability from gene *i* (*g*_*i*_) to metabolite *j* (*m*_*j*_) can be described as


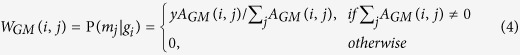


The transition probability from phenotype *i* (*p*_*i*_) to gene *j* (*g*_*j*_) can be defined as


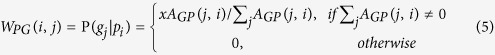


The transition probability from phenotype *i* (*p*_*i*_) to phenotype *j* (*p*_*j*_) can be calculated as follows:


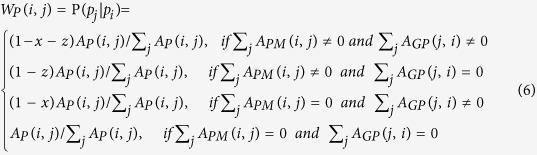


The transition probability from phenotype *i* (*p*_*i*_) to metabolite *j* (*m*_*j*_) can be described as


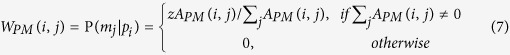


The transition probability from metabolite *i* (*m*_*i*_) to gene *j* (*g*_*j*_) can be defined as


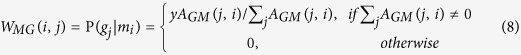


The transition probability from metabolite *i* (*m*_*i*_) to phenotype *j* (*p*_*j*_) can be described as


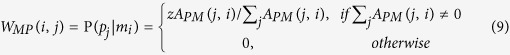


The probability from metabolite *i* (*m*_*i*_) to metabolite *j* (*m*_*j*_) can be computed as


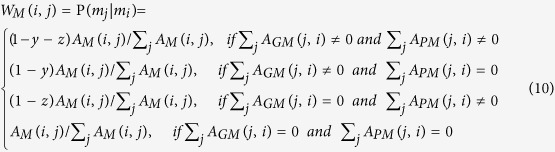


Suppose 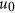, 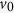, and 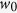 are the initial probabilities of the gene network, phenotype network and metabolite network, respectively. For one phenotype, the seed nodes are composed of i) the phenotype, ii) the corresponding known metabolites and iii) known genes. The initial probability of gene network 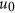 is calculated by assigning equal probability to gene nodes in the gene network, with a sum equal to 1. This means the random walker starts from each of the seed nodes with equal probability. Similarly, the initial probabilities 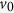 and 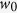 are calculated. Then, the initial probability of the composite network can be defined as 
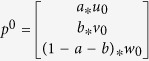
, where *a*, *b* and 1 − *a* − *b* range from 0 to 1, and they denote the importance of the gene network, phenotype network and metabolite network, respectively. After several iterations, the probabilities tend to a steady state, and the steady probability 
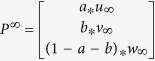
. Then, the candidate metabolites can be ranked according to 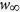. In this study, we set parameter *a* to 0.7 and *x*, *y*, *z*, *a* and *b* to 1/3 unless otherwise noted.

### Assessing the influence of noise in the multi-omics composite network

A new network relation score is obtained by combining the original weight score with noise using equation (1). In this equation, 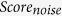 is generated from a uniform distribution U (0, 1), and *σ* is a coefficient that indicates the proportion of noise in the combined score and ranges from 0 to 1.


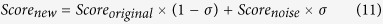


## Results

MetPriCNet prioritizes the disease metabolites by integrating multi-omics information. In this work, a multi-omics composite network is first constructed by integrating information from the genome, phenome, metabolome and interactome (Figure S1). The multi-omics composite network includes three types of nodes (gene, metabolite and phenotype nodes) and six types of interactions (gene-gene, metabolite-metabolite, phenotype-phenotype, gene-metabolite, phenotype-gene, and phenotype-metabolite interactions). This network is composed of 25,629 nodes and 11,926,113 edges (the detailed information is given in Table 1). In this section, we tested the performance of MetPriCNet and compared it with RWR on metabolite network only (PROFANCY). Next, we applied MetPriCNet to prostate cancer and breast cancer to identify high-risk disease metabolites. We also applied MetPriCNet to multiple diseases to explore the metabolic landscape of human disease from a global view.

### Performance of MetPriCNet

**Figure 2:**
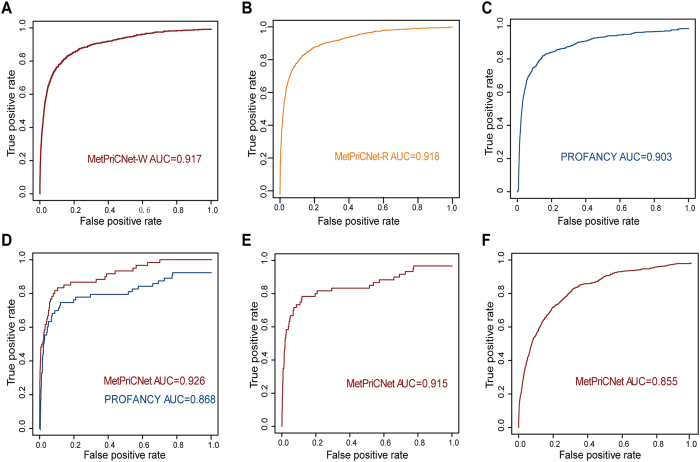
The performance of MetPriCNet method. (**A**)The ROC curve of the overall performance of MetPriCNet in whole-metabolome candidates. (**B**) The ROC curve of the overall performance of MetPriCNet in random candidates. (**C**) The ROC curve of the overall performance of PROFANCY method in whole-metabolome candidates. (**D**) The ROC curves of MetPriCNet method and PROFANCY method in 30 phenotypes with only two known metabolites. Red line indicates MetPriCNet method, blue line represents PROFANCY method. (**E**) The ROC curves of MetPriCNet method in 62 phenotypes with only one known metabolite. (**F**)The ROC curves of MetPriCNet method when only phenotype and corresponding disease genes were used as the seed nodes.

To test the performance of MetPriCNet, we assessed the ability of MetPriCNet to uncover known metabolites. We chose phenotypes that are associated with at least 2 known disease metabolites in the composite network and obtained 87 phenotypes with 602 known disease metabolites and 205 known disease genes. Then, leave-one-out cross-validation was performed for each known disease metabolite. In each run of the cross-validation, one known metabolite was termed as the test metabolite. Firstly, we removed the link between this metabolite and its corresponding phenotype from the composite network. The seed nodes were defined as (1) the target phenotype; (2) the other known metabolites linked to this phenotype; and (3) all known genes linked to this phenotype. We used two types of candidates in MetPriCNet: a random candidate set and a metabolome-wide metabolite set. The former was defined as 100 metabolites, including 99 metabolites that were randomly selected from the metabolite network and the test metabolite. The latter was defined as all metabolites in the metabolite network, excluding the seed metabolites. Then, we performed MetPriCNet and obtained the scores of the candidates. Ideally, the test metabolite should have a high score among the candidates. To evaluate the overall performance, receiver operating characteristic analysis (ROC) was performed by plotting the true positive rate versus the false positive rate at various threshold settings. As shown in Fig. 2A,B, MetPriCNet achieved an AUC (area under the curve) value up to 0.917 in the metabolome-wide metabolite set and of 0.918 in the random candidate set. The high prediction power of MetPriCNet suggests that the strategy using interaction among multi-omics data from the composite network has high efficiency in prioritizing disease metabolites. The AUC result in the two candidate sets showed that the candidate set selection has a slight influence on the prediction power. Thus, we used the metabolome-wide candidate set in the following analyses.

**Figure 3:**
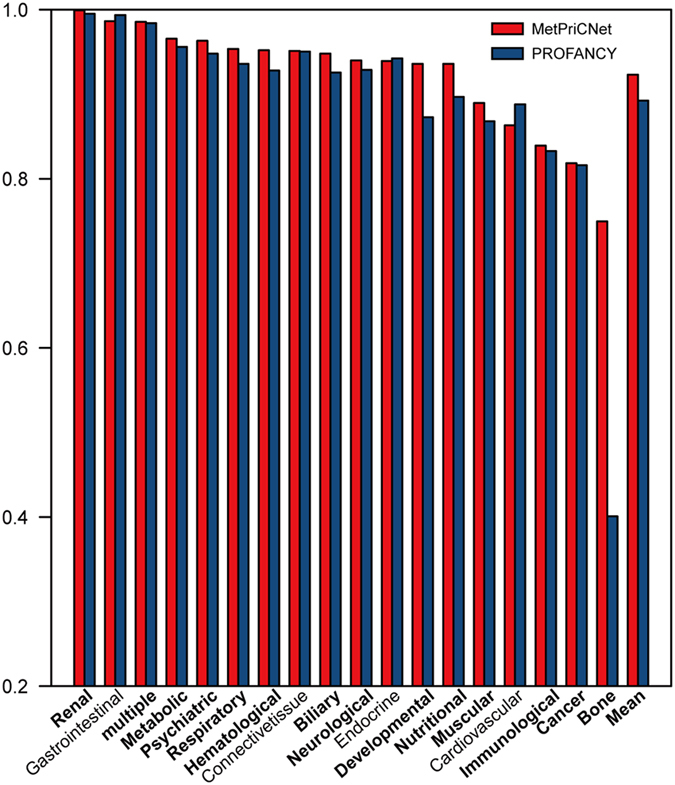
The comparison of performance between MetPriCNet method and PROFANCY method in various disease classes. Red bar indicates MetPriCNet method and blue bar indicates PROFANCY method.

To detect the efficiency of MetPriCNet in various disease classes, we classified 87 phenotypes involved in the composite network into 18 categories according to the study of Goh KI *et al.*^23^. The phenotypes that did not exist in the previous study^23^ were manually assigned to appropriate categories. For each disease class, we performed leave-one-out cross-validation and ROC analysis. As shown in Table S1 and Fig. 3, the mean AUC value of 18 disease classes was 0.903 ± 0.0068, of which, up to 13 (72.2%) disease classes are above 0.9, and 17 (94.4%) disease classes are above 0.77. Especially, the renal disease class had the highest AUC of 0.999. There are 7 known metabolites in the renal disease class, and they are related to two phenotypes (cystinuria disease and autosomal-dominant polycystic kidney disease), of which 6 (85.7%) metabolites ranked top 4 among the metabolome-wide candidates (approximately 3764 metabolites) in the leave-one-out cross-validation. Cystinuria disease is caused by impairment of the transport of cystine, arginine, ornithine, and lysine in the apical membrane of the intestinal epithelium and proximal renal tubule^24^,^25^. Interestingly, these four known disease metabolites (cystine, lysine, arginine and ornithine) ranked top 2 in the leave-one-out cross-validation. These results suggested that MetPriCNet not only had strong overall prediction power, but also performed well in various disease classes.

### Method comparison

To evaluate the advantage of our strategy of integrating multi-omics information, we compared MetPriCNet with random walk with restart on the metabolite network only (PROFANCY)^9^. After performing leave-one-out cross-validation, we found that MetPriCNet could achieve an AUC value up to 0.918, which is higher than that of PROFANCY (0.903) (Fig. 2C), suggesting that MetPriCNet improved the performance by integrating the multi-omics information. We further found that MetPriCNet demonstrated improved performance in 14 of 18 (77.8%) disease classes compared with PROFANCY (Fig. 3). Specially, the AUC value of the development disease class improved from 0.841 to 0.92.

Furthermore, MetPriCNet has another important advantage. When lacking known disease metabolite information, MetPriCNet can use other information, such as the corresponding phenotype and known disease genes, to prioritize the candidate metabolites. To test the performance of MetPriCNet when disease metabolite information is missing, phenotypes that only linked two known metabolites were extracted from the composite network, which left only one metabolite as a seed in the leave-one-out cross-validation. We obtained 30 phenotypes with 60 known disease metabolites and 46 disease genes. Leave-one-out cross-validation was used to test the overall performance of these two methods. For each run, PROFANCY used one metabolite as the seed, and MetPriCNet used seed nodes, including one metabolite, the corresponding phenotype and the disease genes links to the phenotype. We found that the AUC value of MetPriCNet was 0.926 compared to 0.868 for PROFANCY (Fig. 2D). Furthermore, for some diseases for which we did not have any disease metabolite information, PROFANCY lost its efficiency completely. To evaluate the prediction power of MetPriCNet in this situation, we extracted the phenotypes that are only linked to one disease metabolite in the composite network. A total of 62 phenotypes, 128 genes and 62 metabolites were obtained. For each of these phenotypes, we removed the link between the phenotype and the disease metabolite in the multi-omics composite network. The phenotype and its corresponding disease gene were used as seed nodes to run MetPriCNet. The AUC value was 0.915 (Fig. 2E). To further examine the performance of MetPriCNet, we supposed that these phenotypes did not have any known metabolites and performed leave-one-out cross-validation on 87 phenotypes with at least two known disease metabolites, as mentioned above. For each of the 87 phenotypes, we removed all of the links between the phenotype and the known metabolites associated with it. Then, the phenotype and the corresponding disease genes were used as the seed nodes to perform MetPriCNet to obtain the scores of the known metabolites. The resulting AUC value was 0.855 (Fig. 2F).

### Robustness and parameters of MetPriCNet

The composite network was constructed based on six data sources, and false positives were introduced from the different data sets (e.g., PPI network). A previous report on the human PPI network suggested that there was ~14.5% false positives for yeast-two hybrid protein pairs by co-affinity purifications^26^, so we investigated whether MetPriCNet is robust to noise in the data. We introduced noise into the network weight score to assess the robustness of MetPriCNet. The prediction power changes were tested with *σ* varying from 0 to 1 (Figure S2). MetPriCNet achieved an AUC value above 0.79, even if the relation score was perturbed by up to 30% noise. These results indicated that our method is relatively robust to noise.

There are five parameters in MetPriCNet: *α*, *x*, *y*, *z*, *a* and *b. α* is the restart probability in the RWR method. To examine the robustness of this parameter in MetPriCNet, *α* was set to range from 0.1 to 0.9, with intervals of 0.2, and the performance was measured by leave-one-out cross-validation. As shown in Table S2, the performance improved with increasing *α*. When *α* was between 0.5 and 0.9, the performance was stable. *x*, *y*, and *z* are the jumping probability between various networks (for details, see the method section). These parameters reflect the extent of the mutual information between two basic networks. For example, a larger *x* represents more information exchange between genes and phenotypes. To test the effect of these parameters, we assigned extreme values. Additionally, we calculated the overall AUC using MetPriCNet. The results are shown in Table S3. MetPriCNet had better performance when z was above 1/3, which indicates that greater information exchange between the phenotype and metabolite can improve the prioritization. Parameters *a* and *b* indicate the importance of the seed nodes. For instance, *a*=1/3, *b*=1/3 and 1 − *a* − *b* = 1/3 represents an equal-weighted gene network, phenotype network and metabolite network. If the value of *a* is above 1/3, the random walker tends to return to the gene seeds, indicating that the gene network is more important in disease metabolite prioritization. Extreme values were assigned to these parameters to investigate their effects. As illustrated in Table S3, when *b* and 1 − *a* − *b* were above 1/3, MetPriCNet had better performance, which indicated that the phenotype network and metabolite network should be given greater importance.

### Case study

#### Case study 1: Identify prostate cancer risk metabolites using the metabolite profile

We applied MetPriCNet to prostate cancer (PC) to illustrate the efficiency (details of PC shown in the Supplementary Text). After implementation of MetPriCNet, we found that of the 10 metabolites, up to 8 metabolites were well documented as being associated with prostate cancer (Table S4). We also tested the rank of these metabolites in PROFANCY. Three metabolites — glycerol, sucrose and sorbitol — were identified by both MetPriCNet and PROFANCY, indicating that they might be novel risk metabolites in PC. Further inspection showed that these three metabolites all belonged to the galactose metabolism pathway, suggesting that galactose metabolism plays an important role in PC. Seven risk metabolites were ignored by PROFANCY when the rank cutoff was set 10, and all of these metabolites have been reported to be highly associated with PC. The top-ranked metabolite is sarcosine, which was only ranked 21 by PROFANCY. Sarcosine is an amino acid derivative of N-methylglycine, which is involved in amino acid metabolism and methylation processes that are enriched during prostate cancer progression. Sarcosine has been reported as a potentially important metabolic intermediary of cancer cell invasion and aggressiveness^27^,^28^. The second-ranked metabolite is aspartate. Aspartate, which is an essential amino acid, can serve as the four-carbon source of oxaloacetate for citrate synthesis, whose production levels are reduced as a result of altered cellular metabolism and bioenergetics in prostate cancer^29^,^30^. Glutamine is the third-ranked metabolite, though it ranks 37 in PROFANCY. Glutamine has been reported to contribute to many core metabolic tasks in proliferating tumor cells, and prostate cancers cells produce a significant amount of glutamate, which has diagnostic potential to distinguish benign and malignant human prostate tissue^31^,^32^. These three top-ranked metabolites all belong to the arginine, proline and glutamate metabolism pathway, which is highly associated with the proliferation of prostate cancer^33^,^34^. The top seven to ten metabolites — pyrophosphoric acid, cholesterol, uracil and caffeine — which are ignored by PROFANCY, are well documented to be associated with cancer and prostate cancer^35^,^36^,^37^,^38^,^39^,^40^,^41^,^42^,^43^,^44^ (Supplementary Text).

#### Case study 2: Predicting novel risk metabolites of breast cancer in the absence of known disease metabolites

To further demonstrate the advantage of MetPriCNet, we applied the method to breast cancer to predict new disease metabolites. Breast cancer is a genetically complex disorder and is one of the major leading causes of cancer death in women. There are 19 disease genes related with breast cancer in OMIM^15^. However, there is no known disease metabolite information in HMDB^14^. In this condition, PROFANCY completely lost its efficiency. However, MetPriCNet prioritized disease metabolites by taking full advantage of information of known gene and phenotype information. In this analysis, the whole metabolome as candidates and the phenotype of breast cancer and 19 disease genes were used as seeds. The detailed information of the top 5 metabolites predicted by MetPriCNet is listed in Table S5. All of the metabolites are highly associated with the initiation and progression of cancer (Supplementary Text).

**Figure 4:**
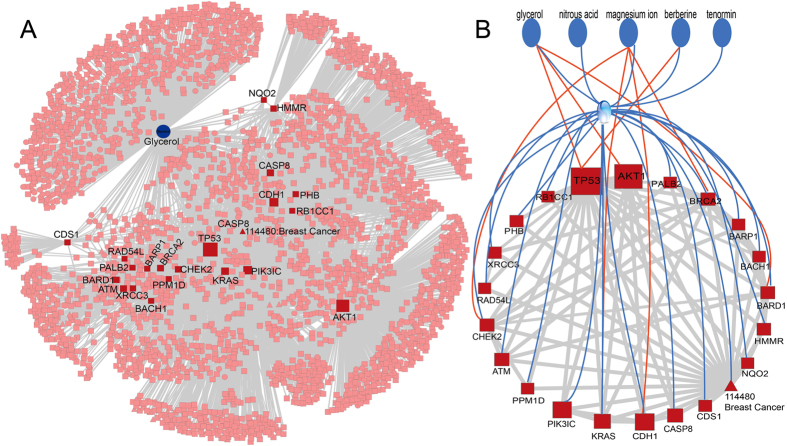
(**A**) The subnetwork of top-ranked risk metabolites, seeds and their first neighbors of breast cancer. Red indicates seed nodes, blue indicates the top-ranked risk metabolite glycerol, and pink indicates the neighbor nodes. Squares indicate genes, triangles indicate phenotypes and circles indicate metabolites. Only interaction scores above 0.6 in the whole composite network are retained. (**B**) The direct and indirect interaction (only two-step neighbors are considered) between the top 5 ranked risk metabolites and seeds in breast cancer. Only interactions with confidence scores above 0.6 are considered. Blue circles represent the top 5 risk metabolites identified by MetPriCNet. Red rectangles represent the seed nodes. Red lines indicate direct interactions, and blue lines indicate indirect interactions.

To further illustrate the intrinsic mode of MetPriCNet, we dissected the top-ranked metabolite glycerol. A subnetwork is extracted from the whole composite network in which only interaction scores above 0.6 are retained (Fig. 4A and Figure S3). In this subnetwork, glycerol connects to 3 seeds directly. Furthermore, there are 869 common neighbors and 2,452 edges between glycerol and the seeds. This strong association with the seeds of breast cancer might imply the reason that metabolite glycerol is ranked first. It is easily noticed that there are three types of nodes among the neighbors, including phenotype nodes, metabolite nodes and gene nodes. This consists with the intrinsic mechanism of MetPriCNet, which can account for the interaction information of every type of node to consider the similarity between the candidate metabolites and the seeds under the global composite network. We further inspected the direct and indirect interaction (we only considered two-step neighbors) between the five top-ranked metabolites and seed nodes. The top-ranked metabolite is expected to have more and stronger direct and indirect interactions with the seeds. Only interactions with a confidence score above 0.6 are extracted. Results shows top-ranked glycerol interacts with three seeds (TP53, AKT1, and BARD1) directly, whereas the third-ranked metabolite, magnesium ion, interacts with four seeds (CDH1, KRAS, CHEK2, CDS1) directly (Fig. 4B). It seems inconsistent with our methods. However, MetPriCNet is based on a global distance measure, which considers not only strong direct interactions but also indirect and weak interactions. Further inspection shows that the three seeds that glycerol directly interacts with more closely interact with the other seeds than that of the magnesium ion. Glycerol indirectly interacts with 18 seeds, and magnesium ion indirectly interacts with 2 seeds. We further infer that nitrous acid is ranked number 2 because it indirectly interacts with these seeds.

Furthermore, we identified the significant KEGG pathways of the top 30 breast cancer candidate metabolites using iSubpathway-GM^45^. These metabolites are significantly enriched in the oxidative phosphorylation pathway, purine metabolism pathway and steroid hormone biosynthesis pathway.

#### Case study 3: A predicted metabolomic landscape of human diseases

**Figure 5:**
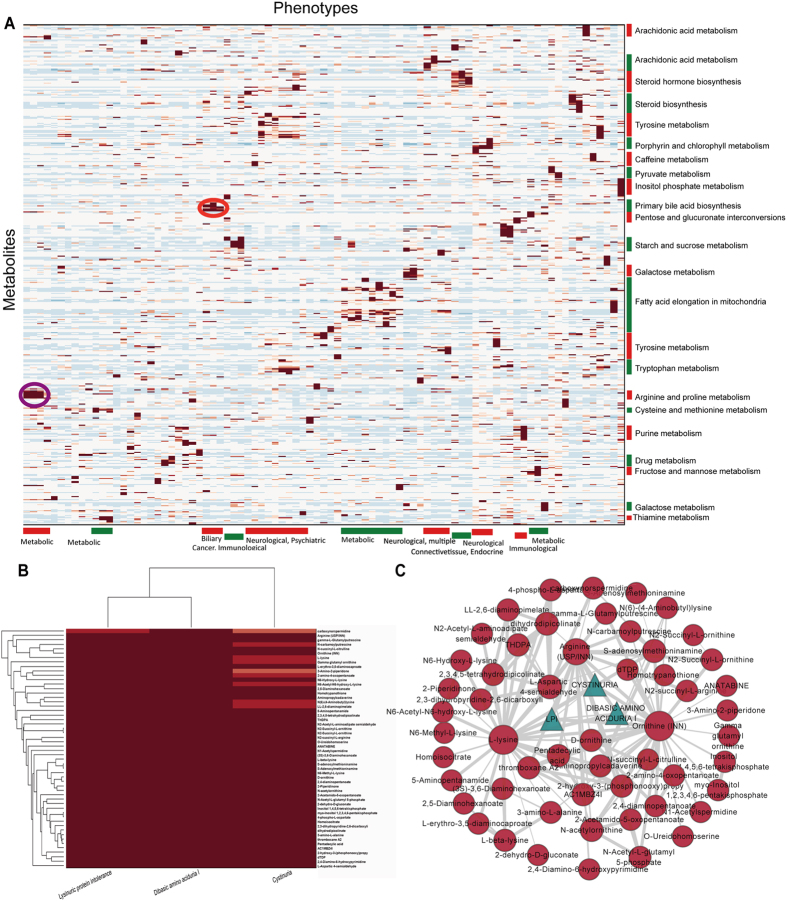
Global view of the predicted landscape of human diseases. (**A**) Hierarchical clustering of the MetPriCNet scores between 87 phenotypes and 3,764 metabolites. The color of each cell represents the MetPriCNet score of a phenotype (column) and a metabolite (row), where red/blue indicates high/low MetPriCNet scores. Phenotype clusters are annotated with enriched disease categories (bottom), and metabolite clusters are annotated with the most enriched pathways of KEGG (right). The purple circled region indicates a module composed of the metabolite set of arginine/proline metabolism involved in a set of metabolic diseases. (**B**) Zoomed-in plot of the purple circled region, involving 3 metabolic diseases and 54 highly related metabolites. (**C**) The sub-network composite of the 3 phenotypes (blue triangles) and 54 highly related metabolites (red circles).

We further applied MetPriCNet to multiple diseases to detect the relationship between diseases and metabolites from a global view. MetPriCNet is used to infer the metabolome-wide molecular basis of the 87 phenotypes and to chart the metabolic landscape of human disease. First, we constructed a score matrix using the MetPriCNet scores between the 87 phenotypes and the 3,764 metabolites. Then, hierarchical clustering was performed to reveal the relation of the phenotypes and metabolites (Fig. 5A). The score matrix indicates the relation between the phenotypes and their corresponding risk metabolites based on genetic and metabolic mechanisms; therefore, the phenotypes that are clustered together tend to share genetic and metabolic overlaps. The phenotype clusters were annotated with enriched disease classes, and the metabolite clusters were annotated with most enriched pathways of KEGG. The result shows that some small modules, including molecular function-related metabolites, are implicated in the set of genetically and metabolically overlapped diseases. For example, the purple circled region in Fig. 5A shows a module enriched with the metabolic disease class and arginine/proline metabolism progression. The zoomed-in plot of this region shows that this module contains 3 diseases and 54 highly related metabolites (Fig. 5B). Furthermore, the metabolites are highly connected in the heterogeneous composite network (Fig. 5C). The metabolites l-lysine, ornithine and arginine, which are the common seeds of these three phenotypes, are highly connected with other risk metabolites. This common metabolic basis contributes to the module. Interestingly, we found that biliary diseases are highly related to primary bile acid biosynthesis (red circle in Fig. 5A). We also noticed that one disease class may be related to multiple biological pathways. For example, metabolic diseases are highly related to many metabolic processes, such as arginine and proline metabolism, galactose metabolism, and fructose and mannose metabolism, which suggests that one disease class may be caused by abnormality of multiple biological processes. Similarly, one biological process can be related to different disease classes, such as tryptophan metabolism being related to neurological and psychiatric diseases, suggesting that different disease classes share some of the same biological processes. The predicted disease metabolite file, which contained 87 disease phenotypes and the top 50 disease metabolites for each disease, has been uploaded to our website (http://www.bio-bigdata.net/MetPriCNet/) for reference.

## Discussion

The biological system can be presented as a composite network with multi-omics functional interaction information. Genetic or environmental perturbations influence the transcriptional level and cell metabolism and are reflected in abnormal phenotypes. Therefore, integrating multi-omics information will help us infer disease risk metabolites. Based on this idea, we developed a powerful network-based method, MetPriCNet, for candidate metabolite prioritization, which considers the full topology of the weighted composite network by integrating the genome, phenome, metabolome and interactome information. We assessed the performance of MetPriCNet by conducting leave-one-out cross-validation on 87 phenotypes with 602 metabolites. MetPriCNet achieved an overall AUC up to 0.918. When assessing the performance of MetPriCNet on 18 disease classes, the result showed that 4 disease classes achieved an AUC value over 0.95 and a mean AUC of 0.903 ± 0.0068. Compared with PROFANCY, MetPriCNet performed well not only at overall level but also for various disease classes. MetPriCNet achieved better performance in 77.8% of the disease classes. Notably, MetPriCNet can be applied to diseases without known metabolite information. We tested the performance of MetPriCNet on phenotypes that are linked to two known metabolites and phenotypes that are only linked to one known metabolite. After removing known disease genes and metabolites, MetPriCNet can still achieve an AUC value of 0.855. This result might benefit from interactions between the phenotype seed and other elements of the network. The results show that MetPriCNet performs well under both conditions. However, the performance of PROFANCY decreases and even loses its efficiency, which suggests that no less than one seed (gene, metabolite or phenotype) for each phenotype is needed for MetPriCNet to work. We now address why PROFANCY outperformed MetPriCNet (Fig. 3) for 4 of the 18 disease classes. The ROC curve is created by plotting the true positive rate versus false positive rate at various threshold settings based on the prioritization list of the metabolites. If the known disease metabolites tend to rank at the top of the prioritization list, the AUC value is higher. For the cases that PROFANCY outperformed MetPriCNet, maybe due to that top-ranked metabolites of the prioritization list are novel disease-related metabolites that currently have not been reported in experiments. Actually, these metabolites were closely connected with the seed nodes (known disease genes, metabolites and associated phenotypes) in the composite network, which indicates that they were highly associated with the target disease. We further searched the literature for the top-ranked metabolites in the prioritization list for these four disease classes. We found that, after the accomplishment of this manuscript, Khan *et al.* demonstrated that hypertension and associated cardiovascular pathophysiological changes induced by angiotensin II occur through the release of arachidonic acid^46^. Arachidonic acid is one of the top-ranked metabolites in the prioritization list of the leave-one-out cross-validation experiment, which further indicates the potential of MetPriCNet for the prediction of novel disease metabolites. In addition, metabolites are closely related to diseases, whereas genes interact with metabolites that are involved in the metabolic reaction. Thus, it is reasonable to predict disease metabolites by integrating phenome, genome and metabolome data. The integrated novel information (phenome and genome data) can contribute to the prioritization of disease metabolites by information flow transfer in the multi-omics composite network and may be helpful for the discovery of novel disease metabolites. We have demonstrated that MetPriCNet can effectively predict disease metabolites without known disease metabolite knowledge and its potential for predicting novel disease metabolites.

The success of MetPriCNet can be attributed to the joint power of several aspects. First, the composite network was composed of six networks, integrating multi-omics information from genome, phenome, metabolome and interactome. MetPriCNet takes advantage of multi-omics information. The information from various omics levels is highly connected and interacted, and although there is a lack of some information, MetPriCNet can still use other information to prioritize the candidate metabolites. Second, an extended random walk method is developed to capture the global multi-omics information. This method guarantees that the candidate metabolites were ranked based on the interaction information in the entire composite network, rather than merely the local environment. There are some limitations of MetPriCNet. The random walk model usually uses the rank number to decide which candidate is more likely to be a real element. The higher the rank of the candidate is, the more likely it is to be a real element^9^,^13^,^47^,^48^,^49^. To test the statistical significance of the top ranked metabolites, we performed 1000 times permutations while preserving the degrees of the multi-omics composite network and prioritized candidate metabolites based on the generated the same number of seed nodes. We calculated p value for random metabolites as probability of observing them with higher score than our predicted score (detail can be found in supporting information Table S6 and S7). Scientists could select their own disease risk metabolites based on rank number, statistial significance and literature research. MetPriCNet depends on the topology of the composite network, the incompleteness and bias of the interactions of the multi-omics network limits the performance. Although MetPriCNet performs well with an incomplete network, its performance could be further improved after more accurate and complete reconstructions of the composite network. In future study, the strategy of multi-omics composite network can be used in various fields in biomedicine, such as disease, drug and target discovery.

## Additional Information

**How to cite this article**: Yao, Q. *et al.* Global Prioritization of Disease Candidate Metabolites Based on a Multi-omics Composite Network. *Sci. Rep.* **5**, 17201; doi: 10.1038/srep17201 (2015).

## Supplementary Material

Supplementary Information

Supplementary Table S6

Supplementary Table S7

## Figures and Tables

**Table 1:** The statistic information of the composite network.

Statistics of the composite Network	Node	Edge
gene network	16785	1515370
metabolite network	3764	74667
phenotype network	5080	10140046
gene-metabolite association network	12342 genes, 3278 metabolite	192763
phenotype-gene association network	1886 phenotypes, 1715 genes	2603
phenotype-metabolite association network	149 phenotypes, 388 metabolites	664
All	25629	11926113
