# Supplementary material for: Global Prioritization of Disease Candidate Metabolites Based on a Multi-omics Composite Network
Source: Sci Rep. 2015 Nov 24;5:17201. doi: 10.1038/srep17201 (PMC4657017; doi:10.1038/srep17201)
Supplement: Supplementary Information [file srep17201-s1.doc]

**Supporting information**

Global Prioritization of Disease Candidate Metabolites Based on a Multi-omics Composite Network

Qianlan Yao^1,+^, Yanjun Xu^1,+^, Haixiu Yang^1^, Desi Shang^1^, Chunlong Zhang^1^, Yunpeng Zhang^1^, Zeguo Sun^1^, Xinrui Shi^1^, Li Feng^1^, Junwei Han^1^, Fei Su^1^, Chunquan Li^1,2^,* and Xia Li^1^,*

^1^ College of Bioinformatics Science and Technology, Harbin Medical University, Harbin, 150081, China

^2^ School of Medical Informatics, Daqing Campus, Harbin Medical University, 39 Xinyang Road, Harbin 163319, China

***Case study 1: Identify prostate cancer risk metabolites using the metabolite profile***

In this case, we applied MetPriCNet to prostate cancer (PC), which is the second leading cause of cancer-death in men. First, we obtained the GC/LC-MS metabolic profiles which contained hundreds of named metabolites across 38 prostate tissues (16 benign adjacent prostates; 12 clinically localized prostate cancers) [^1^](#_ENREF_1). This high throughput profiles quantitatively detected 175 metabolites, of which 138 metabolites in our composite network, there were 6 known PC metabolites and the remaining 132 metabolites were considered as candidates. The phenotype MIM number 176807 and 14 known disease genes from OMIM database and 31 known disease metabolites from HMDB database were used as seed nodes.

Pyrophosphoric acid, ranked seventh, could form bisphosphonates, which exert direct anti-tumour effects on a variety of human tumour cell lines including prostate cancer. The eighth ranked metabolite is cholesterol, ranked 66 in PROFANCY. It belonged the steroidal lipid, is an essential membrane component of animal cells. It functions as a mediator of cell proliferation, membrane dynamics, inflammation and steroid genesis, providing multiple avenues for this lipid to contribute to prostate cancer progression. High circulating cholesterol increases risk of aggressive prostate cancer, while cholesterol lowering strategies may confer protective [^4-7^](#_ENREF_4). Uracil ranked nine in MetPriCNet, it is significantly elevated upon disease progression from benign to PC to Mets in the dataset we used and could potentially serve as biomarkers for progressive disease . Caffeine is a major component in coffee, and some animal studies have reported that caffeine can stimulate and suppress tumours, according to the species and the phase of administration [^9^](#_ENREF_9). These results demonstrated that MetPriCNet can effectively identify disease risk metabolites by integrating omics data.

***Case study 2: Predicting novel risk metabolites of breast cancer in the absence of known disease metabolites***

The top one ranked glycerol has long been known to play fundamental roles in several vital physiological processes and is an important intermediate of energy metabolism. Some studies have suggested that the glycerol treatment to tumours enhanced growth delay in tumour mice. The second ranked metabolite is nitrous Acid. It has been known that nitrous acid can damage nucleic acids by deamination or nitration and play an important role in cancers . The third ranked metabolite is magnesium ion, which is essential for integrin-ligand binding and is important for cell adhesion and cancer metastasis [^14^](#_ENREF_14). Berberine is the forth ranked metabolite. It is a natural alkaloid with significant antitumor activities against many types of cancer cells. It can reduce the metastatic potential of highly metastatic breast cancer cells and may be a useful adjuvant therapeutic agent in the treatment [^15^](#_ENREF_15). It also has been reported to inhibit the proliferation of MCF-7 breast cancer cells through a mitochondria and caspase dependent apoptotic pathway and may serve as a potential naturally occurring compound for breast cancer therapy [^16^](#_ENREF_16). The fifth ranked is tenormin, also known as atenolol. It is a beta-1 adrenergic blocker, which can inhibit cell migra­tion and metastasis has been tested and replicated using in vivo and in vitro models [^17^](#_ENREF_17). In the study by Melham-Bertrandt et al. suggested that patients receiving β‑blockers (β1-selective β‑blockers, metoprolol and atenolol) showed a significant improve­ment in 3‑year relapse-free survival and can reduce tumor recurrence [^18^](#_ENREF_18).

**Supplementary Figures**


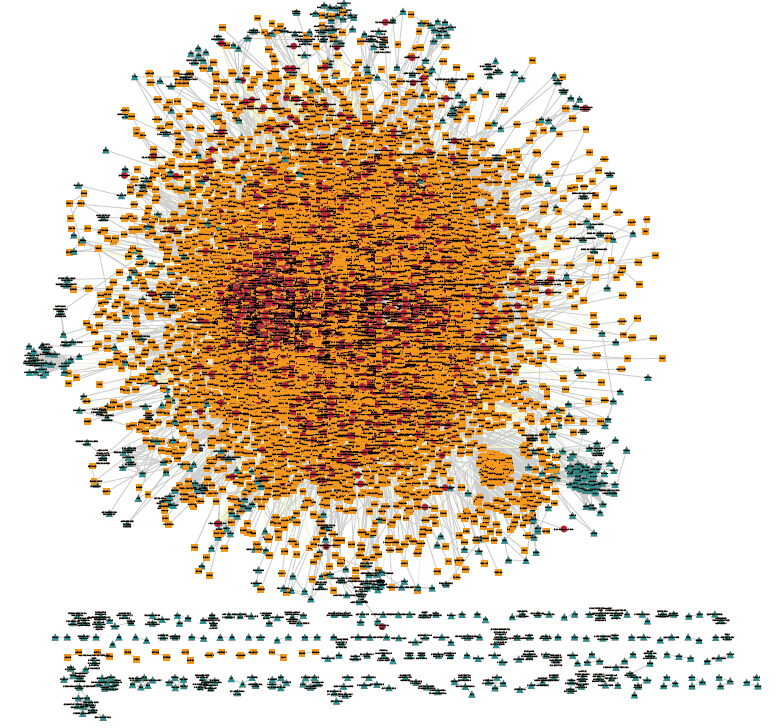


Figure S1. The [sketch map](app:ds:sketch  map) of multi-omics composite network. We only show the weight score of edges of multi-omics composite network not less than 0.8. Where the blue triangle indicates phenotype node, orange squares represent genes node, red circle node represents metabolic child nodes. The thickness of edges represents their weight scores.


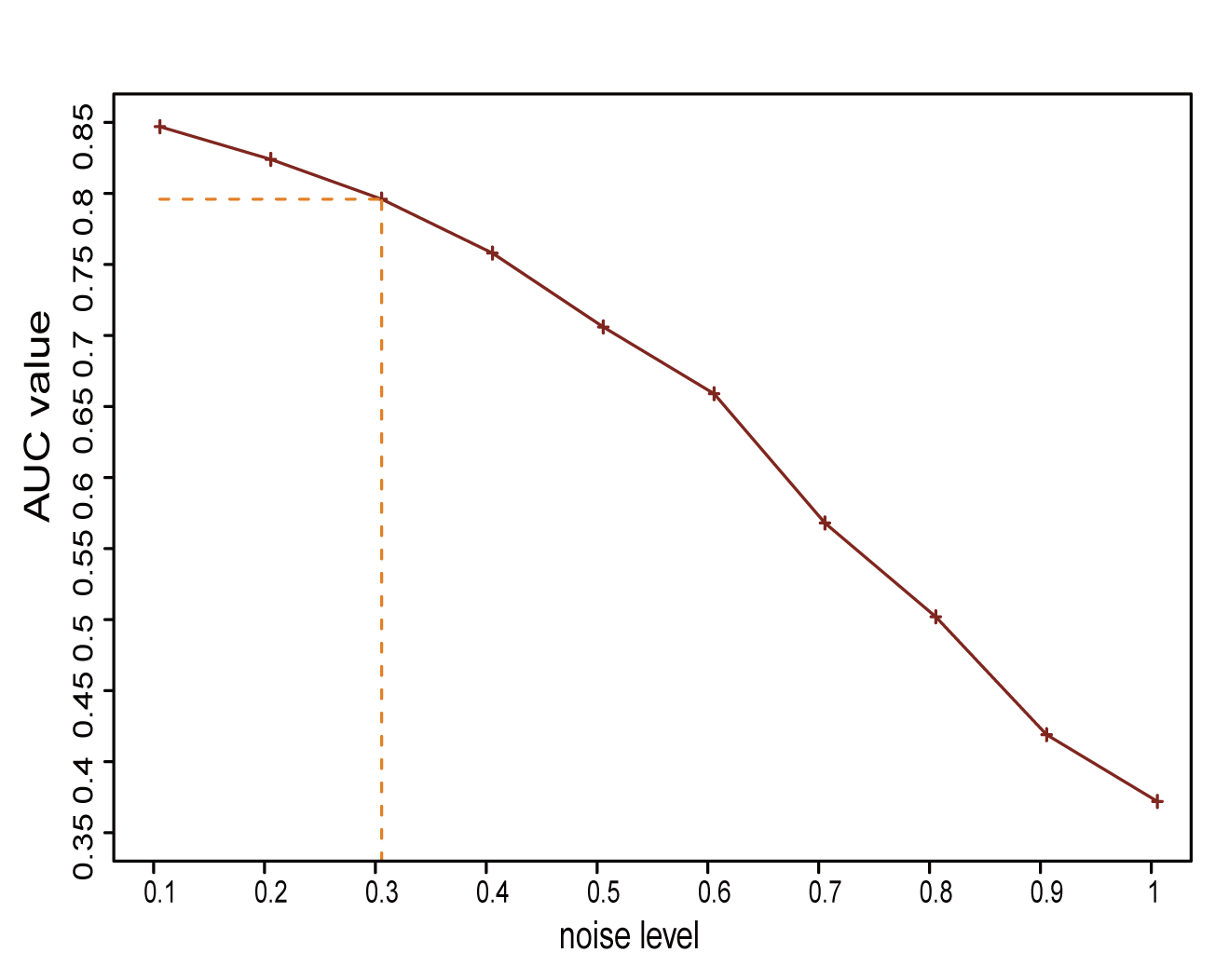


Figure S2. The robust performance of MetPriCNet in various noise levels.


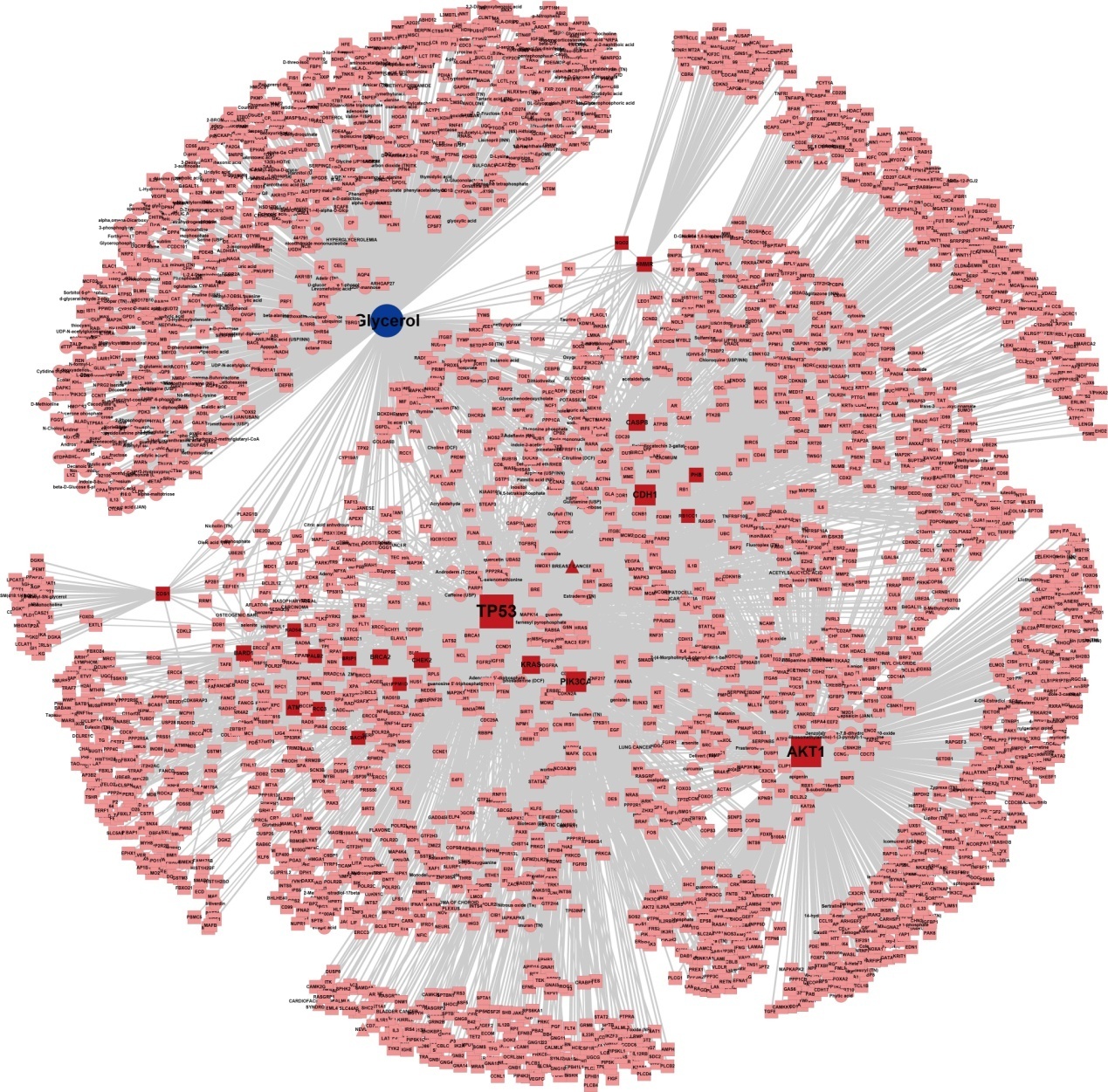


Figure S3. The subnetwork of top-ranked risk metabolite, seeds and their first neighbors of breast cancer. Red indicates seed nodes, blue indicates the top 1 ranked risk metabolite glycerol, and pink indicates their neighbour nodes. Squares indicate gene, triangles indicate phenotype and circle indicates metabolite. Only interaction score above 0.6 in the whole composite network were retained.

**Supplementary Tables**

**Table S1.** The comparison of performance of MetPriCNet and PROFANCY in 18 disease classes. Gray shadow indicates disease classes in which the AUC value of MetPriCNet is higher than that of PROFANCY method

| Disease Class | No.of  Phe | No.of NDM | No.of NDG | AUC of  MetPriCNet  (WholeM) | AUC of  MetPriCNet  (Random) | AUC of  PROFANCY  (WholeM) | AUC of  PROFANCY  (Random) |
| --- | --- | --- | --- | --- | --- | --- | --- |
| Biliary | 1 | 9 | 0 | 0.935 | 0.935 | 0.907 | 0.904 |
| Bone | 1 | 2 | 5 | 0.687 | 0.689 | 0.251 | 0.25 |
| Cancer | 6 | 72 | 49 | 0.773 | 0.778 | 0.770 | 0.774 |
| Cardiovascular | 1 | 13 | 13 | 0.829 | 0.839 | 0.86 | 0.859 |
| Connective  tissue | 1 | 11 | 7 | 0.939 | 0.933 | 0.938 | 0.937 |
| Developmental | 1 | 2 | 3 | 0.92 | 0.919 | 0.841 | 0.823 |
| Endocrine | 3 | 37 | 31 | 0.924 | 0.923 | 0.928 | 0.931 |
| Gastrointestinal | 1 | 2 | 3 | 0.983 | 0.977 | 0.992 | 0.984 |
| Hematological | 2 | 11 | 1 | 0.94 | 0.94 | 0.91 | 0.908 |
| Immunological | 3 | 8 | 1 | 0.799 | 0.801 | 0.791 | 0.775 |
| Metabolic | 41 | 172 | 47 | 0.957 | 0.957 | 0.945 | 0.945 |
| multiple | 2 | 9 | 9 | 0.982 | 0.988 | 0.98 | 0.98 |
| Muscular | 1 | 4 | 1 | 0.862 | 0.87 | 0.835 | 0.852 |
| Neurological | 13 | 170 | 28 | 0.925 | 0.926 | 0.911 | 0.91 |
| Nutritional | 2 | 18 | 17 | 0.92 | 0.918 | 0.871 | 0.874 |
| Psychiatric | 4 | 39 | 18 | 0.954 | 0.953 | 0.935 | 0.933 |
| Renal | 2 | 7 | 2 | 0.999 | 0.999 | 0.994 | 0.995 |
| Respiratory | 2 | 16 | 15 | 0.942 | 0.943 | 0.92 | 0.92 |
| Mean | 87 | 602 | 250 | 0.903 | 0.904 | 0.865 | 0.864 |

No.of Phe: Number of phenotype contained in certain disease class; No.of NDM: Number of known disease metabolites; No.of NDG: Number of known disease genes; WholeM: AUC value in metabolome-wide metabolite set; Random: AUC value of in random candidate set; Mean: The mean value of 18 disease classes.

**Table S2.** The effect of parameter$\alpha$.

| Parameter | Case1 | Case8 | Case9 | Case10 | Case11 |
| --- | --- | --- | --- | --- | --- |
| $\alpha$ | 0.1 | 0.3 | 0.5 | 0.7 | 0.9 |
| AUC | 0.886 | 0.908 | 0.915 | 0.917 | 0.916 |

**Table S3.** The effect of parameter $x$,$y$,$z$,$a$ and $b$.

| Parameter | Case4 | Case5 | Case6 | Parameter | case1 | case2 | case3 |
| --- | --- | --- | --- | --- | --- | --- | --- |
| $x$ | 0.1 | 0.1 | 0.8 | $a$ | 0.1 | 0.1 | 0.8 |
| $y$ | 0.1 | 0.8 | 0.1 | $b$ | 0.1 | 0.8 | 0.1 |
| $z$ | 0.8 | 0.1 | 0.1 | $1-a-b$ | 0.8 | 0.1 | 0.1 |
| AUC | 0.924 | 0.897 | 0.914 | AUC | 0.92 | 0.92 | 0.901 |

**Table S4.** Top 10 metabolites of PC identified by MetPriCNet and their ranks in PROFANCY method. “*”represent metabolites only identified by MetPriCNet.

| Metabolite  Names | PubChem  Id | MetPriCNet  Score | MetPriCNet  pvalue | MetPriCNet  Rank | PROFANCY  Score | PROFANCY  Rank | Reference |
| --- | --- | --- | --- | --- | --- | --- | --- |
| sarcosine* | 1088 | 0.002041 | 0.0087 | 1 | 0.000619 | 21 |  |
| aspartate* | 5960 | 0.000485 | 0.0169 | 2 | 0.000663 | 14 | [^21-23^](#_ENREF_21) |
| glutamine* | 5961 | 0.000457 | 0.0158 | 3 | 0.000371 | 37 | [^24-26^](#_ENREF_24) |
| glycerol | 753 | 0.000329 | 0.0202 | 4 | 0.000919 | 6 |  |
| sucrose/maltose | 5988 | 0.000321 | 0.0214 | 5 | 0.001803 | 1 | - |
| sorbitol | 5780 | 0.000269 | 0.0247 | 6 | 0.001751 | 2 | - |
| pyrophosphoric acid* | 1023 | 0.000241 | 0.0268 | 7 | 0.00073 | 12 |  |
| cholesterol* | 5997 | 0.000233 | 0.0275 | 8 | 0.000141 | 66 | [^4-7^](#_ENREF_4) |
| uracil* | 1174 | 0.000229 | 0.0304 | 9 | 0.000635 | 18 |  |
| caffeine* | 2519 | 0.000221 | 0.0300 | 10 | 0.000172 | 59 | [^9^](#_ENREF_9) |

**Table S5.** Top 5 breast cancer risk metabolites identified by MetPriCNet.

| Metabolite  Names | PubChem  Id | MetPriCNet  Score | MetPriCNet  pvalue | MetPriCNet  Rank | Reference |
| --- | --- | --- | --- | --- | --- |
| glycerol | 753 | 0.002259 | 0.0001 | 1 |  |
| nitrous acid | 24529 | 0.001862 | 0.0005 | 2 |  |
| magnesium ion | 888 | 0.001133 | 0.0010 | 3 | [^27^](#_ENREF_27) |
| berberine | 2353 | 0.000822 | 0.0017 | 4 |  |
| tenormin | 2249 | 0.000782 | 0.0019 | 5 |  |

**REFERENCES**

1 Sreekumar, A. *et al.* Metabolomic profiles delineate potential role for sarcosine in prostate cancer progression. *Nature* **457**, 910-914, doi:nature07762 [pii]

10.1038/nature07762 (2009).

2 Saad, F. & Schulman, C. C. Role of bisphosphonates in prostate cancer. *Eur Urol* **45**, 26-34, doi:S0302283803005177 [pii] (2004).

3 Todenhofer, T. *et al.* Altered expression of farnesyl pyrophosphate synthase in prostate cancer: evidence for a role of the mevalonate pathway in disease progression? *World J Urol* **31**, 345-350, doi:10.1007/s00345-012-0844-y (2013).

4 Krycer, J. R. & Brown, A. J. Cholesterol accumulation in prostate cancer: a classic observation from a modern perspective. *Biochim Biophys Acta* **1835**, 219-229, doi:S0304-419X(13)00003-6 [pii]

10.1016/j.bbcan.2013.01.002 (2013).

5 Pelton, K., Freeman, M. R. & Solomon, K. R. Cholesterol and prostate cancer. *Curr Opin Pharmacol* **12**, 751-759, doi:S1471-4892(12)00117-8 [pii]

10.1016/j.coph.2012.07.006 (2012).

6 Thysell, E. *et al.* Metabolomic characterization of human prostate cancer bone metastases reveals increased levels of cholesterol. *PLoS One* **5**, e14175, doi:10.1371/journal.pone.0014175 (2010).

7 Hager, M. H., Solomon, K. R. & Freeman, M. R. The role of cholesterol in prostate cancer. *Curr Opin Clin Nutr Metab Care* **9**, 379-385, doi:10.1097/01.mco.0000232896.66791.62

00075197-200607000-00007 [pii] (2006).

8 Miyake, H., Hara, I., Yamazaki, H. & Eto, H. Clinical outcome of oral uracil/tegafur (UFT) therapy for patients with hormone refractory prostate cancer. *Oncol Rep* **14**, 673-676 (2005).

9 Shafique, K. *et al.* Coffee consumption and prostate cancer risk: further evidence for inverse relationship. *Nutr J* **11**, 42, doi:1475-2891-11-42 [pii]

10.1186/1475-2891-11-42 (2012).

10 Yuki, K. *et al.* Sensitization by glycerol for CDDP-therapy against human cultured cancer cells and tumors bearing mutated p53 gene. *Apoptosis* **9**, 853-859, doi:5384705 [pii]

10.1023/B:APPT.0000045795.21285.a1 (2004).

11 McCarty, M. F. Prospects for glycerol-rescued hypoglycemia as a cancer therapy. *Med Hypotheses* **56**, 286-289, doi:10.1054/mehy.2000.1156

S0306-9877(00)91156-3 [pii] (2001).

12 Chen, H. J., Hsieh, C. J., Shen, L. C. & Chang, C. M. Characterization of DNA--protein cross-links induced by oxanine: cellular damage derived from nitric oxide and nitrous acid. *Biochemistry* **46**, 3952-3965, doi:10.1021/bi0620398 (2007).

13 Maeda, H. & Akaike, T. Nitric oxide and oxygen radicals in infection, inflammation, and cancer. *Biochemistry (Mosc)* **63**, 854-865 (1998).

14 Lymburner, S., McLeod, S., Purtzki, M., Roskelley, C. & Xu, Z. Zinc inhibits magnesium-dependent migration of human breast cancer MDA-MB-231 cells on fibronectin. *J Nutr Biochem*, doi:S0955-2863(12)00206-9 [pii]

10.1016/j.jnutbio.2012.07.013 (2012).

15 Kuo, H. P. *et al.* Berberine, an isoquinoline alkaloid, inhibits the metastatic potential of breast cancer cells via Akt pathway modulation. *J Agric Food Chem* **60**, 9649-9658, doi:10.1021/jf302832n (2012).

16 Patil, J. B., Kim, J. & Jayaprakasha, G. K. Berberine induces apoptosis in breast cancer cells (MCF-7) through mitochondrial-dependent pathway. *Eur J Pharmacol* **645**, 70-78, doi:S0014-2999(10)00756-9 [pii]

10.1016/j.ejphar.2010.07.037 (2010).

17 Schuller, H. M. Beta-adrenergic signaling, a novel target for cancer therapy? *Oncotarget* **1**, 466-469, doi:101102 [pii] (2010).

18 Melhem-Bertrandt, A. *et al.* Beta-blocker use is associated with improved relapse-free survival in patients with triple-negative breast cancer. *J Clin Oncol* **29**, 2645-2652, doi:JCO.2010.33.4441 [pii]

10.1200/JCO.2010.33.4441 (2011).

19 Cavaliere, B. *et al.* Sarcosine as a marker in prostate cancer progression: a rapid and simple method for its quantification in human urine by solid-phase microextraction-gas chromatography-triple quadrupole mass spectrometry. *Anal Bioanal Chem* **400**, 2903-2912, doi:10.1007/s00216-011-4960-0 (2011).

20 Baum, C. E., Price, D. K. & Figg, W. D. Sarcosine as a potential prostate cancer biomarker and therapeutic target. *Cancer Biol Ther* **9**, 341-342, doi:11310 [pii] (2010).

21 Levin, Y. S. *et al.* Methods for metabolic evaluation of prostate cancer cells using proton and (13)C HR-MAS spectroscopy and [3-(13)C] pyruvate as a metabolic substrate. *Magnetic resonance in medicine : official journal of the Society of Magnetic Resonance in Medicine / Society of Magnetic Resonance in Medicine* **62**, 1091-1098, doi:10.1002/mrm.22120 (2009).

22 Costello, L. C. & Franklin, R. B. Prostate epithelial cells utilize glucose and aspartate as the carbon sources for net citrate production. *Prostate* **15**, 335-342 (1989).

23 Costello, L. C. & Franklin, R. B. The clinical relevance of the metabolism of prostate cancer; zinc and tumor suppression: connecting the dots. *Molecular cancer* **5**, 17, doi:10.1186/1476-4598-5-17 (2006).

24 Hensley, C. T., Wasti, A. T. & DeBerardinis, R. J. Glutamine and cancer: cell biology, physiology, and clinical opportunities. *The Journal of clinical investigation* **123**, 3678-3684, doi:10.1172/JCI69600 (2013).

25 Daye, D. & Wellen, K. E. Metabolic reprogramming in cancer: unraveling the role of glutamine in tumorigenesis. *Seminars in cell & developmental biology* **23**, 362-369, doi:10.1016/j.semcdb.2012.02.002 (2012).

26 Hahn, P. *et al.* The classification of benign and malignant human prostate tissue by multivariate analysis of 1H magnetic resonance spectra. *Cancer Res* **57**, 3398-3401 (1997).

27 Lymburner, S., McLeod, S., Purtzki, M., Roskelley, C. & Xu, Z. Zinc inhibits magnesium-dependent migration of human breast cancer MDA-MB-231 cells on fibronectin. *J Nutr Biochem* **24**, 1034-1040, doi:S0955-2863(12)00206-9 [pii]

10.1016/j.jnutbio.2012.07.013 (2013).
